# Time bomb

**DOI:** 10.1038/s44319-024-00273-9

**Published:** 2024-09-25

**Authors:** Howy Jacobs

**Affiliations:** Tampere University, Tampere, Finland

**Keywords:** Economics, Law & Politics, Science Policy & Publishing

## Abstract

Can the scientific literature act as a guide to how social media might self-regulate?

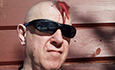

The current and growing furore over the use of social media to disseminate lies, fake news, hateful opinions and even incitement to violence has placed all those who cherish both free speech and the rule of law in a quandary. Should the internet and its various platforms be subject to regulation? If so, how should it be policed and by whom? Who draws the line between truth and lies, between appeals for justice and calls for riots on the streets? Can any such restriction be reconciled with traditional notions of freedom of expression? Is it the thin end of a wedge that leads to the arbitrary exercise of power and oppressive dictatorship? Is the destructive influence of smartphone culture, especially on the young, leading to an intolerable proliferation of anxiety, depression, self-harm and even suicide? Plus bullying and overt criminality. Are young people being distracted from their studies and from normal social interactions by participating in an online world that simply isn’t real? Are social media platforms already in the grip of corporate and state actors that seek to manipulate and ultimately control us all (Albert, 2024)?

The issues raised seem intractable and pose an obvious and growing threat to humanity. Nobody has yet come up with a satisfactory solution, while the problems seem to be multiplying.

But we have a robust and largely successful model to guide us, namely the scientific literature. Although it is not ‘perfect’, those of us involved in scientific publishing—which includes just about all active and even some inactive experimental scientists—believe what we read and write in the research literature to be honest, accurate and objective. This does not occur by magic. Scientific publishers, journal editors and authors abide by a rigorous code of conduct, set out explicitly in various formats or just implicit in the norms of ethical academic behaviour. We have extensive guidelines and tools to facilitate self-regulation within these norms. Where norms are broken, or where cases of doubt arise, we are expected to defer to independent bodies. In Finland, where I am based, we have an independent panel of experts, nominated by the scientific community to advise and adjudicate on all matters concerning research integrity (TENK, https://tenk.fi/en/tenk). Similar bodies in many other countries ensure compliance with ethical standards by identifying and investigating cases of egregious misconduct in science and in scientific reporting. Based on their enquiries they issue decisions and recommendations that are almost invariably respected and followed, in some cases to the level of criminal proceedings.

Yes, there are a few ‘scientific outlaws’ who remain outside this framework of self-regulation, and whose work and writings are propagated by scammers and other miscreants. But we have a widespread awareness of individuals and organisations in this grey zone and how to spot them. They are systematically shunned by the overwhelming majority of scientists and scientific institutions. Despite minor differences, the code of conduct to which we all subscribe is, essentially, universal. It is mostly immune to the influence of the varied political systems under which we live. It applies in astrophysics or sociology as much as in neuroscience or cell biology.

What is preventing broader society from establishing and operating an equivalent system of self-regulation, backed up by independent adjudication, to prevent the spread of harmful content on the internet? The scientific literature is, of course, tiny compared with the published output of humanity as a whole. Even this is dwarfed by the informal and ephemeral writings of billions of individuals on social media. But operational principles should be similar. In science, the costs of self-regulation and oversight mechanisms are born by scientific publishers, hence indirectly by research funders and institutions. But there is no reason why social media platforms and internet providers cannot do likewise. They could voluntarily devote 1% of their enormous profits to defining and agreeing ethical norms and devising mechanisms to enforce them. And for those operators who stand outside of such a system, the public can vote with its feet and simply blacklist them.

Many will undoubtedly reply to this suggestion that the profit motive is simply too strong for the media and ‘tech’ companies to move in this direction. But the major scientific publishers are also commercial corporations, and this has not prevented them from exercising proper responsibility in their field of operations, with little if any legislative compulsion.

The alternative seems to be that, over time, we will all cease to believe what we read. In which case we will have nothing but myths and legends on which to base our view of the world. The time bomb of misinformation and disinformation will have exploded, taking with it our ability to think for ourselves. This might even happen in science. But for now, fake science has only a tiny constituency, and has remained in the shadows, largely because the system of self-regulation and independent oversight actually works. I think it’s time for the rest of humanity to catch up.

## Supplementary information

Peer Review File
