# Supplementary material for: Time bomb
Source: EMBO Rep. 2024 Sep 25;25(11):4627–8. doi: 10.1038/s44319-024-00273-9 (PMC11549457; doi:10.1038/s44319-024-00273-9)
Supplement: Supplementary file 1 — Peer Review File [file 44319_2024_273_MOESM1_ESM.pdf]

EMBO reports

Time Bomb

Howard Jacobs

Corresponding author(s): Howard Jacobs (howard.jacobs@tuni.fi)

**Review Timeline:**	Submission Date:	17th Sep 24
	Accepted:	17th Sep 24

Editor: Holger Breithaupt

Transaction Report: This article type is not peer reviewed.
